# An analysis of unplanned postoperative admissions to the intensive care units at different hospitals across Hamad Medical Corporation in Qatar

**DOI:** 10.5339/qmj.2025.35

**Published:** 2025-06-11

**Authors:** Hashaam Ghafoor, Yasser Hammad, Ali Bel Khair, Osman Ahmed, Ekambaram Karunakaran, Hamed Mohamed Elgendy, Wael Mohammad Khalaf, Shaikh Nissaruddin, Hossam Mohamed Algallie, Tarek Anwar Ahmed Tageldin, Ashok Kandasamy, Gulzar Hussain, Amber Naz, Mariam Ali Karrar El Obied, Sanjeev Dharamchand Jain, Lamia Mahmoud Mohamed Tawfik, Walaa Mohamed Sayed Hassan, Nazeer Ahmed, Mohammed Huzain K. Bukhari

**Affiliations:** 1 Department of Anesthesia Hamad Medical Corporation, Doha, Qatar *Email: hghafoor@hamad.qa

**Keywords:** Unplanned admission, postoperative, intensive care unit

## Abstract

**Background:**

An unplanned intensive care admission (UIA) after elective surgery is a clinical indicator of patient safety and outcomes. Furthermore, it reflects both surgery- and anesthesia-related complications. The overall rate of UIA ranges from 0.28% to 2.2%. UIA is linked with higher rates ofmorbidity and mortality in surgical patients. Thus, understanding the factors leading to UIAs could improve the quality of patient care. In this study, we aimed to determine the rate and reasons for UIA following elective surgeries in public facilities in Qatar.

**Methods:**

UIA was defined as an admission to the intensive care unit (ICU) within 72 hours of anesthesia that was not anticipated during the pre-anesthesia assessment phase. A multicenter audit was conducted from January 1, 2021, to December 31, 2021, across five public hospitals in Qatar. UIA was identified from the electronic preoperative and postoperative anesthetic assessment notes and intraoperative notes.

**Results:**

Among the 2,087 ICU admissions, 42 (2.0%) were UIAs. Among the 42 patients, 57.1% were males, and the mean age was 41.83 ± 12.95 years. Most patients (64.3%) were classified as American Society of Anesthesiologists status II. The mean length of ICU stay was 2.60 ± 2.45 days. Most of the UIAs were surgery-related (54.8%), followed by anesthesia-related (26.2%) and medical-related (16.6%).

**Conclusion:**

The rate of UIA in our study was 2%, corresponding to the wide range of incidence reported in the literature. The causes of UIA are multiple; however, our study showed that the rate of anesthesia-related UIAs was 26.2%, which is less than in most previous studies.

## Introduction

The intensive care unit (ICU) is primarily involved in the care of patients with injuries and life threatening, severe, recoverable, and critical illnesses. It is an organized system for providing specialized medical and nursing care using specialized equipment and medications.^1^ More than 310 million surgeries are being performed worldwide every year.^2^ More than 40% of ICU admissions are related to postoperative care.^3^ The decision to admit a patient to the ICU is assessed preoperatively and based on the severity of the patient’s comorbidities, risks of the surgical procedure, and institutional policy.^4^ However, some patients may require an unplanned ICU admission (UIA) due to unexpected adverse events or abrupt deterioration during the perioperative period.^5^

Patients admitted to the ICU following the development of complications related to surgery tend to have worse outcomes than those who are admitted to the ICU preemptively.^6^ UIA can be used as a tool to identify medical errors and unanticipated adverse events during the perioperative period. Thus, UIA is a significant safety indicator among surgical patients.^7^The term UIA was initially utilized in the United States of America as a surrogate indicator for patient safety. Subsequently, it has been utilized in several other countries worldwide.^8^ The rate of UIA reportedly ranges from 0.28% to 2.2%.^5^ This variation in the incidence of UIA may be attributed to the difference in the UIA definition, inclusion criteria, or institutional practice between the studies. The rate of anesthesia-related UIA among patients varies from 0.04% to 0.45%.^9^

Postoperative UIA represents a significant group of patients in whom the need for intensive care develops only within the immediate perioperative period. UIA may be attributed to emergencies or adverse events that occur perioperatively or postoperatively, indicating that complications are related to both patient illness and patient care. These patients are considered a high-risk population with a mortality rate of approximately 15%.^10^

Admission to the ICU may increase the length of hospital stay after surgery.^11^ Patients with a UIA experience longer ICU stays and higher mortality rates than those with planned ICU admissions.^8^ UIA increases the family members’ anxiety and the workload of healthcare workers, which places a significant financial burden on the healthcare system. Thus, analyses of unplanned admissions facilitate the identification of high-risk patients and the ideal allocation of resources, which enhances patient safety.^12^ Additionally, UIA is associated with adverse outcomes and is considered an indicator of the quality of the entire perioperative period.^13^ Hamad Medical Corporation (HMC) is the cornerstone of the country’s healthcare system, serving as Qatar’s largest public healthcare provider.^14^ HMC includes different hospitals such as Hamad General Hospital (HGH; 603 beds), Al Wakra Hospital (AWH; 325 beds), Women’s Wellness and Research Center (WWRC; 240 beds), Hazm Mebaireek General Hospital (HMGH; 118 beds), Al Khor Hospital (AKH; 115 beds), and Ambulatory Care Center (ACC; 66 beds). These facilities offer a broad range of services in almost all the domains of healthcare. To date, no study has been conducted to determine the rate of and causes of UIA in these healthcare facilities. Further analysis of the risk factors of UIA could enhance healthcare quality and reduce the incidence of UIA. Thus, in this study, we aimed to determine the rate of UIA and identify surgery-, anesthesia-, medical-, and patient-related risk factors for UIA following elective surgeries.

## Methods

This prospective multicenter study was approved by the Corporate Anesthesia Department’s Quality, Patients’ Safety, and Performance Monitoring Committee (No: CA00026; dated 05 October 2020). The study was conducted between January 1, 2021, and December 31, 2021, at WWRC, AKH, AWH, ACC, and HGH. Any admission to the ICU within 72 hours of an elective surgical procedure without ICU admission being considered during the pre-anesthetic assessment was defined as UIA. Only UIAs due to surgical, anesthetic, and medical complications were assessed.

Preoperatively, at the reception of the operating theater, the planned postoperative ICU admission status was recorded and cross-checked. The following data were collected from the electronic preoperative and postoperative anesthesia assessment notes as well as intraoperative notes: demographic data including body mass index (BMI), American Society of Anesthesiologists (ASA) physical status score, reason for ICU admission, anesthesia-related factors, surgery-related factors, medical-related factors, and length of ICU stay.

Data were analyzed using SPSS (version 25.0). Both quantitative and qualitative variables were descriptively analyzed. The quantitative variables such as age, BMI, and length of ICU stay are presented as means and standard deviations (SD). The qualitative variables such as sex, intubation during ICU admission, specialty-wise ICU admission, and surgical, medical, and anesthesia causes for UIA are presented as frequencies and percentages.

## Results

There were a total of 2,087 ICU admissions during the study period. Of these, 42 (2%) were UIA (Table 1). Because the study period overlapped with COVID-19, all COVID-19-related admissions were excluded. ICU admissions from HMGH were not included, as nearly all of them were COVID-19-related.

Of the 42 patients with UIAs, 35 (83.3%) were evaluated in the pre-anesthesia clinic; 23 patients (54.8%) were <40 years old, and 19 patients (45.2%) were >40 years old. The mean age of these patients was 41.83 ± 12.958 years.

Of the 42 patients with UIAs, 24 (57.1%) were males and 18 (42.9%) were females; 1 patient (2.4%) was underweight (BMI < 18.5%), 9 (21.4%) had a normal weight (BMI 18.5–24.9), 17 (40.5%) were overweight (BMI 25.0–29.9), and 15 (35.7%) were obese (BMI > 30) (Table 2).

Most of the patients with UIAs were classified as ASA II (*n* = 27; 64.3%), followed by ASA III (*n* = 8; 19.0%) and ASA I (*n* = 7; 16.7%). Most patients with UIAs were not intubated before being moved to the ICU (*n* = 30; 71.4%). Only 12 patients (28.6%) were intubated before the UIA, of which 23.8% were kept intubated after the procedure for postoperative ventilation in the ICU, and 4.8% were extubated in the OR but needed reintubation before being moved to the ICU. The length of hospital stay was 1–2 days in 26 patients (61.9%), 3–4 days in 10 patients (23.8%), and >4 days in 6 patients (14.3%) (Table 2).

**Figure 1:**
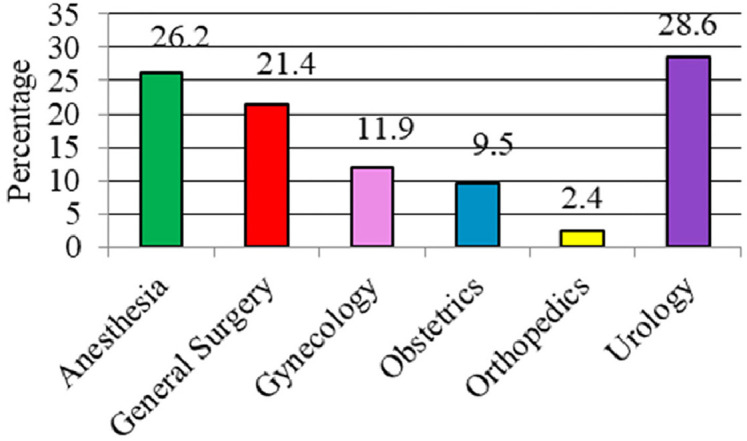
Unplanned intensive care unit admissions according to specialty across the five facilities that were audited during the year 2021.

**Figure 2:**
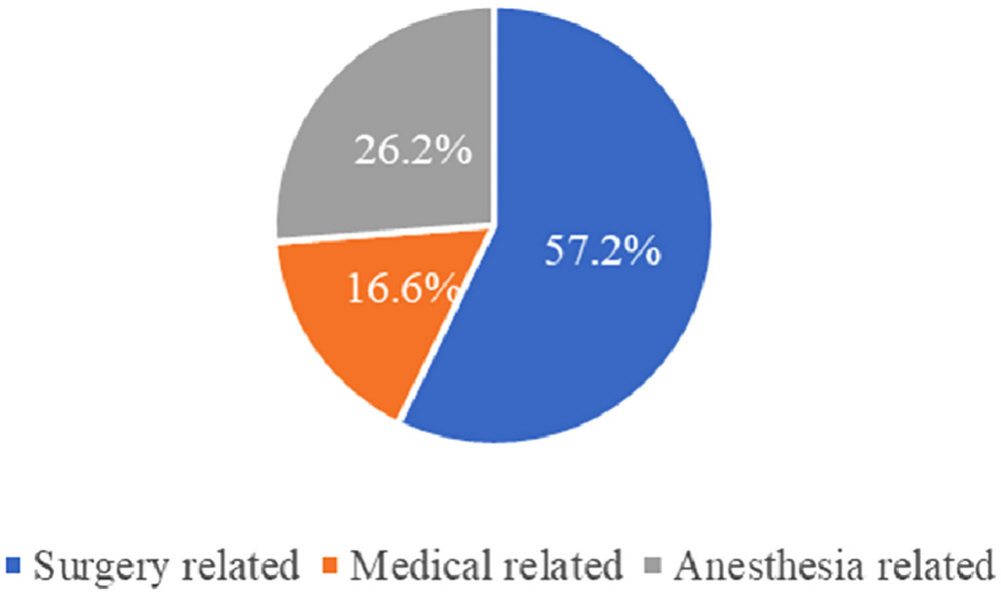
Percentage of surgery-, anesthesia-, and medical-related unplanned intensive care unit admissions following elective surgeries across the five audited facilities during the year 2021.

According to surgical specialties, most UIAs were from urology surgery (*n* = 12; 28.6%), followed by anesthesia (*n* = 11; 26.2%), general surgery (*n* = 9; 21.4%), gynecology (n = 5; 11.9%), obstetrics (*n* = 4; 9.5%), and orthopedics (*n* = 1; 2.4%) (Figure 1). Overall, the UIAs were surgery-, anesthesia-, and medical-related in 24 (57.2%), 11 (26.2%), and seven (16.6%) patients, respectively (Figure 2).

The most common cause for surgery-related UIAs was bleeding (*n* = 9; 21.4%), followed by urosepsis (*n* = 7; 16.7%), and sepsis (*n* = 3; 7.1%). The infrequent causes were embolism, anastomosis leak, long surgery, nerve injury, and organ injury (*n* = 1 each; 2.4%). The most common cause for medical-related UIA was arrhythmias (*n* = 5; 11.8%), followed by generalized fits (*n* = 1; 2.4%) and high blood pressure (*n* = 1; 2.4%) (Table 3).

The most common cause for anesthesia-related UIA was difficult intubation or multiple intubation attempts (*n* = 2; 4.8%), followed by anaphylactic reactions (*n* = 2; 4.7%). The infrequent causes were aspiration, reintubation due to aspiration in the operating room (OR), delayed recovery from anesthesia, malignant hyperthermia, negative-pressure pulmonary edema, pain control, and reintubation in the OR (2.4% each) (Table 4).

## Discussion

During our study period, there were 2,087 ICU admissions across five hospitals. Of these admissions, 42 were UIAs (2.0%). The overall rate of UIA varies over a wide range from 0.686% to 82.8%.^8,15^ The rate of UIA in our study was lower than that reported in previous studies: 23.2%,^3^ 81.2%,^4^ 82.8%,^8^ 29.8%,^16^ 24.1%,^17^ 9.1%,^18^ and 35.0%.^19^ The high incidence in these studies may be attributed to small sample sizes, inadequate perioperative planning, poor risk stratification, and insufficient patient optimization. Furthermore, the varied inclusion criteria, which encompassed children, older adults, patients with diabetes, and patients who underwent bariatric surgery, may have also contributed to the increased incidence rates.^3,4,8,16–19^ The incidence rate in our study was comparable to those of Okafor (5.2%; 26 of the 497 ICU admissions) and Emerson et al. (5.1%; 36 of the 712 ICU admissions), both having the same inclusion criteria.^20,21^ The rate of UIA in our study is higher than what was reported by Cao et al. at the Affiliated Hospital of Jining Medical University, China, who reported a UIA rate of 0.686% (81 of the 11,807 ICU admissions) following neurosurgery.^15^ This lower rate of UIA may be attributed to the inclusion criteria of only cerebral surgery patients.

Age is a factor that may increase the probability of ICU admission. Qatar has a relatively young population. Over 70% of the population consists of expatriate young male workers, and approximately 83% of them are within the age range of 15–65 years.^22^ In our study, the mean age of the patients was 41.83 ± 12.96 years (Table 2). In contrast, in a study performed in India by Singh et al., the mean age of the patients was 53.18 ± 16.56 years.^1^ In another Indian study conducted by Bansal et al., the mean age of the patients was 52.44 ± 17.85 years, which is higher than the age of our patients.^3^ A substantial proportion of India’s population is between the ages of 15 and 64 years, and these individuals are known as the working-age population. For more than two decades, >60% of the Indian population has been within the working-age group, and this proportion peaked (66%) in 2018.^23^ This discrepancy highlights the potential differences in patient demographics between different regions and might have resulted in different findings.

Gender distribution is also evident in our study. Over 70% of Qatar’s population consists of males.^22^ This may have accounted for more males than females in our study (57.1% vs. 42.9%; Table 2). This higher proportion of males to females is also evident in a Turkish study by Uzman et al. in which more than half of the patients were male 57.6% compared to 42.4% females.^24^

In our study, the mean BMI of the patients with UIA was 29.08 ± 6.81 (Table 2), which is slightly lower than what was reported by Sukhonthamarn et al. and Phoowanakulchai et al., the mean BMI of the patients was 33.5 ± 8.75^25^ and 25.6 ± 7.00,^26^ respectively. In our study, a majority of the patients (64.3%) were classified as ASA II, followed by ASA III (19.0%) and ASA I (16.7%) (Table 2), which is comparable to the study conducted by Tsuboi et al., where most of the patients were classified as ASA II (41.0%), followed by ASA III (32.0%) and ASA I (27.0%).^12^ Moreover, in our study, 28.6% of the patients who were categorized as UIA were already intubated before being transferred to the ICU (Table 2). However, in the study by Mitchell et al., 46.0% of the patients were intubated at the time of ICU admission.^27^

Patients with UIA experience prolonged hospital stays and significantly higher morbidity and mortality rates. These patients are considered high-risk, with a mortality rate as high as 15%.^10^ In our study, most of the patients (61.9%) were admitted to the ICU for 1–2 days; 23.8% and 14.3% of the patients were admitted for 3–4 days and >4 days, respectively (Table 2). These results are comparable to those of a study by Singh et al., in which most of the patients (50.0%) with UIAs were admitted to the ICU for up to 2 days, followed by 3–4 days (32.0%) and >4 days (18.0%).^1^

Our results indicated that the majority of patients who were categorized as UIA were urology patients (28.6%), general surgery (21.4%), gynecology (11.9%), obstetrics (9.5%), and orthopedic (2.4%) (Figure 1). However, a study carried out by Singh and collaborators reported that most UIA patients were in GI surgery (40.0%), followed by orthopedics (26.0%), urology (12.0%), obstetrics and gynecology (8.0%), general surgery (6.0%), ENT (4.0%), and neurosurgery (4.0%).^1^ Our findings revealed that the overall specialty-wise UIA due to surgery and medical was (73.8%) while anesthesia-related UIAs were only 26.2% (Figure2). This finding is similar to a study by Meziane et al., in which the rate of surgery-related ICU admissions was higher than that of anesthesia-related admissions (64.0% vs. 36.0%).^13^

In our study, the surgery-related UIAs (57.2%) were due to air embolism, anastomosis leak, bleeding, long surgery, nerve injury, organ injury, sepsis, or urosepsis. The medical-related UIAs (16.6%) were due to arrhythmias, generalized fits, or high blood pressure. The anesthesia-related UIAs (26.2%) were due to an anaphylactic reaction, aspiration, reintubation due to aspiration in the OR, delayed recovery from anesthesia, difficult intubation, multiple intubation attempts, malignant hyperthermia, negative-pressure pulmonary edema, pain control and reintubation in the OR (Tables 3 and 4). In the study by Tsuboi et al., the incidence of anesthesia-related UIAs was 55.0%, and their causes were severe post-extubation stridor, postoperative hypoxia, anaphylaxis, drug error, insufficient fluid administration, postoperative apnea, prolonged emergence, uncontrollable emergency delirium, aspiration, acute exacerbation, and bronchial asthma.^12^ The incidence of surgery-related UIAs was 22.0%, and their causes were surgical complications, excessive bleeding, and prolonged surgery.^12^ The causes for the medical-related UIAs 11.0% in the study by Tsuboi et al. were exacerbation of a preexisting medical condition and a newly identified medical condition in the postoperative period. They reported that 12.0% of the UIAs were due to a combination of surgery-, medical-, and anesthesia-related factors.^12^ In the study by Mitchell et al., anesthesia- and surgery-related UIAs accounted for 49.5% and 37.6% of all the UIAs, respectively.^27^ Additionally, 12.9% of the UIAs were due to a combination of surgery- and anesthesia-related factors. Mitchell et al. also demonstrated that the anesthesia-related UIAs were due to postoperative respiratory failure, post-extubation apnea, cardiac arrest, bronchospasm and laryngospasm during induction, seizures, allergic reaction, postoperative cardiopulmonary distress, difficult extubation, postoperative hypertension, difficult intubation, and vomiting during induction. They also reported that the surgery-related UIAs were due to bleeding, hypovolemic shock, small bowel perforation, and migration of the inferior vena cava umbrella filter into the left pulmonary artery.^27^ In the study by Meziane et al., the anesthesia-related UIAs were attributed to cardiac shock, anaphylactoid drug reaction, acute atrial fibrillation, and malignant hyperthermia. However, the surgery-related UIAs were attributed to excessive bleeding in the OR.^13^ In the study by Hezkial et al., the UIAs were attributed to bleeding, hypotension, seizure, bradycardia, infection, and hypoxia/tachypnoea.^28^ In their study, Katori et al. attributed the UIAs to hypotension, arrhythmia, anaphylaxis, bleeding, delayed emergence agitation, and airway obstruction.^5^ Haller et al. attributed the UIAs in their study to hemorrhage, apnea, arrhythmia, angina, complications of intubation, damage to internal organs, pneumothorax, seizures, and uncontrolled pain.^29^

## Conclusion

In conclusion, our analysis of UIA in participating public hospitals in Qatar during the year 2021 revealed an incidence of 2%. Our results are comparable to previous studies regarding surgery- and medical-related factors. In our study, anesthesia-related factors contribute to 26.2% of UIA, which is lower than most of the previously reported figures. We believe that the lower rate of anesthesia-related factors reported in our study could be attributed to the better screening of patients during preoperative assessment. Future research may investigate the development of a standard screening tool for the likelihood of postoperative ICU admission to combine anesthesia, surgical, medical, and patient factors.

## Conflicts of interest

None.

## Figures and Tables

**Table 1.** Number of ICU admissions and UIAs across the five audited hospitals.

**Hospital**	**Total ICU admissions**	**UIAs, *n* (%) percentage**
HGH	827	9 (1.10%)
ACC/WWRC	216	20 (9.26%)
AWH	286	9 (3.15%)
AKH	758	4 (0.53%)
Total	2,087	42 (2.01%)

ICU: intensive care unit, UIA: unplanned ICU admission.

**Table 2.** Characteristics of the study patients (*n* = 42), ASA and intubation status, length of stay in ICU (days), ICU admission specialty-wise.

**Characteristic**	**Number, *n* (%) percentage**
**Seen in pre-anesthesia clinic**	
Yes	35 (83.3%)
No	7 (16.7%)
**Age, years**	
<40	23 (54.8%)
>40	19 (45.2%)
Mean + SD	41.83 ± 12.958
**Sex**	
Male	24 (57.1%)
Female	18 (42.9%)
**BMI**	
<18.5	1 (2.4%)
18.5–24.9	9 (21.4%)
25.0–29.9	17 (40.5%)
>30	15 (35.7%)
Mean + SD	29.08 ± 6.814
**ASA status**	
I	7 (16.7%)
II	27 (64.3%)
III	8 (19.0%)
**Intubation at the time of ICU admission**	
Yes	12 (28.6%)
No	30 (71.4%)
**Length of ICU stay, days**	
1–2	26 (61.9%)
3–4	10 (23.8%)
>4	6 (14.3%)
Mean + SD	2.60 ± 2.450
**ICU admission according to specialty**	
Anesthesia	11 (26.2%)
General surgery	9 (21.4%)
Gynecology	5 (11.9%)
Obstetrics	4 (9.5%)
Orthopedics	1 (2.4%)
Urology	12 (28.6%)

SD: standard deviation, ASA: American Society of Anesthesiologists, ICU: intensive care unit.

**Table 3.** Subgroup analysis of the surgery- and medical-related factors for UIAs.

**Causes for UIA**	**Number, n (%) percentage**
**Surgery-related factors**	**24 (57.2%)**
Air embolism	1 (2.4%)
Anastomosis leak	1 (2.4%)
Bleeding	9 (21.4%)
Lengthy surgery	1 (2.4%)
Nerve injury	1 (2.4%)
Organ injury	1 (2.4%)
Sepsis	3 (7.1%)
Urosepsis	7 (16.7%)
**Medical-related factors**	**7 (16.6%)**
Arrhythmia	5 (11.8%)
Generalized fits	1 (2.4%)
High BP	1 (2.4%)

UIA: unplanned ICU admission, BP: blood pressure.

**Table 4.** Anesthesia-related causes for UIA following elective surgeries.

**Causes for UIA**	**Number, n (%) percentage**
**Anesthesia-related**	**11 (26.2%)**
Anaphylactic reaction	2 (4.7%)
Aspiration	1 (2.4%)
Reintubation due to aspiration in the OR	1 (2.4%)
Delayed recovery from anesthesia	1 (2.4%)
Difficult intubation and multiple intubation attempts	2 (4.8%)
Malignant hyperthermia	1 (2.4%)
Negative-pressure pulmonary edema	1 (2.4%)
Pain control	1 (2.4%)
Reintubation in the OR	1 (2.4%)

UIA: unplanned ICU admission, OR: operating room.
